# Transcriptional Regulation of PP2A-Aα Is Mediated by Multiple Factors Including AP-2α, CREB, ETS-1, and SP-1

**DOI:** 10.1371/journal.pone.0007019

**Published:** 2009-09-14

**Authors:** He-Ge Chen, Wen-Jun Han, Mi Deng, Jichao Qin, Dan Yuan, Jin-Ping Liu, Ling Xiao, Lili Gong, Songping Liang, Jian Zhang, Yun Liu, David Wan-Cheng Li

**Affiliations:** 1 Department of Biochemistry & Molecular Biology, College of Medicine, University of Nebraska Medical Center, Omaha, Nebraska, United States of America; 2 Key Laboratory of Protein Chemistry and Developmental Biology of Education Ministry of China, College of Life Sciences, Hunan Normal University, Changsha, Hunan, China; 3 Department of Ophthalmology & Visual Sciences, College of Medicine, University of Nebraska Medical Center, Omaha, Nebraska, United States of America; University of Hong Kong, Hong Kong

## Abstract

Protein phosphatases-2A (PP-2A) is a major serine/threonine phosphatase and accounts for more than 50% serine/threonine phosphatase activity in eukaryotes. The holoenzyme of PP-2A consists of the scaffold A subunit, the catalytic C subunit and the regulatory B subunit. The scaffold subunits, PP2A-Aα/β, provide a platform for both C and B subunits to bind, thus playing a crucial role in providing specific PP-2A activity. Mutation of the two genes encoding PP2A-Aα/β leads to carcinogenesis and likely other human diseases. Regulation of these genes by various factors, both extracellular and intracellular, remains largely unknown. In the present study, we have conducted functional dissection of the promoter of the mouse PP2A-Aα gene. Our results demonstrate that the proximal promoter of the mouse PP2A-Aα gene contains numerous cis-elements for the binding of CREB, ETS-1, AP-2α, SP-1 besides the putative TFIIB binding site (BRE) and the downstream promoter element (DPE). Gel mobility shifting assays revealed that CREB, ETS-1, AP-2α, and SP-1 all bind to PP2A-Aα gene promoter. In vitro mutagenesis and reporter gene activity assays reveal that while SP-1 displays negative regulation, CREB, ETS-1 and AP-2Aα all positively regulate the promoter of the PP2A-Aα gene. ChIP assays further confirm that all the above transcription factors participate the regulation of PP2A-Aα gene promoter. Together, our results reveal that multiple transcription factors regulate the PP2A-Aα gene.

## Introduction

Protein phosphorylation and dephosphorylation are the most important regulatory mechanisms modulating functions of more than one third of the total cellular proteins [1]. Protein serine/threonine phosphatase 2A (PP-2A) is a major eukaryotic phosphatase, regulating many different functions including metabolism, DNA replication, transcription, RNA splicing, translation, cell cycle progression, cell senescence and apoptosis, cell transformation, morphogenesis, development, and neurotransmission [1]–[6].

PP-2A exists in both core enzyme and holoenzyme within cells [6]–[7]. The core enzyme consists of a 65 kDa scaffolding protein known as A subunit tethering a 36 kDa catalytic C subunit [7]. Both A and C subunits exist in α and β isoforms encoded by different genes [6]. The full specific activity towards a certain substrate of PP-2A core enzyme is achieved through binding of a variable regulatory subunit to form the heterotrimeric holoenzyme [6]. So far, at least 16 genes have been identified encoding 4 subfamilies of the regulatory subunits: B, B′, B″ and B′″ [7]–[10].

The scaffold subunit of PP-2A bears unique structure features. The 65 kDa protein (both α and β isoforms) contains 15 tandem repeats with a conserved 39-residue sequence known as a Huntington-elongation-A subunit-TOR (HEAT) motif [11]–[13], which is organized into an extended, L-shaped molecule [14]. The catalytic subunit recognizes one end of the elongated scaffolding subunit by interacting with the conserved ridges of HEAT repeats 11–15, while the regulatory subunit contact the scaffold by interacting with the conserved HEAT repeats 1 to 10 [7], [15]–[16].

The functional importance of the PP-2A scaffold subunit is derived from the two important observations. First, mutations in both α and β isoforms of the scaffolding subunit result in compromised binding to the regulatory or catalytic subunit of PP-2A. As a result, the functional scaffold subunits are diminished or substantially reduced and the specific PP-2A activity is compromised [17]. A variety of primary human tumors including lung and colon cancers are associated with the mutations of the scaffold subunits [18]–[22]. Secondly, deregulation of the scaffold subunit expression leads to distinct downregulation of PP-2A activity, causing brain tumors [23]. A reduced expression of PP2A-Aα was also observed in the breast cancer cells MCF-7 [24]. In addition, changed expression of the scaffold subunits may contribute to altered activity of PP-2A, which is implicated in multiple ocular diseases including retina degeneration [25] and cataract [26].

At present, however, very little is known about the regulation of expression of the PP-2A scaffold subunits. To get insight into the regulation of PP2A-Aα/β expression, we have cloned the promoter regions of the genes encoding the scaffold subunits for mouse PP-2A. Here, we report the functional dissection of the PP2A-Aα gene promoter through sequential deletion, in vitro mutagenesis, gel mobility shifting, reporter gene activity and ChIP assays. Our results demonstrate that numerous transcription factors including ETS-1, CREB, AP-2α and SP-1 regulate the PP2A-Aα gene promoter.

## Material and Methods

### Cell culture

Embryonic human lens epithelial cells (FHL124 line) and mouse lens epithelial cells (αTN4-1) were kindly provided by Dr. John Reddan (Oakland University) and Dr. Paul Russell (University of California at Davis), respectively. Human retinal pigment epithelial cells [27] were obtained from ATCC. All cells were cultured in monolayers at 37°C and 5%CO_2_ in Eagle's MEM containing 10% FBS, 2 mM L-glutamine, and 1% penicillin and streptomycin as previously described [27]–[29].

### Molecular cloning of the PP2A-Aα promoter and creation of A1 to A6 deletion mutants

The genomic DNAs used for cloning of the PP2A-Aα promoter were extracted from the muscle tissue of the adult mice. Isolation of the mouse muscle tissue was described before [30]. Seven different primers (Table 1) were designed for PCR reactions using mouse genomic DNA as template. The amplified mouse PP2A-Aα promoter (A1) or the truncated promoter fragments (A2 to A6) were separately inserted into pGL3-basic, a background luciferase reporter gene vector at Xho I and Hind III restriction sites using standard molecular cloning techniques as described before [31].

**Table 1:** OLIGO PRIMERS USED FOR CLONING OF PP2A-Aα PROMOTOR.

Primer name	Oligo primer sequence
A1-F	5′-CTCGAGCTCGAGCACTCGAGCCCTGTTGATGT –3′
A2-F	5′-CTCGAGCTCGAGAATGGTCCAAGAAGGCACTG –3′
A3-F	5′-CTCGAGCTCGAGATGGCTATGCCTTCTGTTCG –3′
A4-F	5′-CTCGAGCTCGAGCCCACCTTCTTCCCTTTCAT –3′
A5-F	5′-CTCGAGCTCGAGTATGAGGCAGAGGTCCATCC –3′
A6-F	5′-CTCGAGCTCGAGACATCTCATTCGTCCGGCCA –3′
Primer-R	5′**-AAGCTTAAGCTT**CTTGGCTCCCTGGCGTTTCTATC –3′

Note: For the convenience, two restriction enzyme recognition sites: Xho I (CTCGAGCTCGAG) and Hind III **(-AAGCTTAAGCTT**) sites were added to the Primer-F and Primer-R, respectively.

### Western Blot Analysis

Western blot analysis was conducted as previously described [32]. Total proteins were extracted from ARPE-19 and FHL124 cells. Fifty µg of total proteins were used for each lane of loaded sample. The protein blots were blocked with 5% milk in TBS buffer overnight at 4°C and then incubated for 1 h or overnight with antibodies against, PP2A-Aα, CREB and AP-2α (Cell Signaling Technology, MA), ETS-1 and SP-1 (Santa Cruz Biotechnology Inc, CA), and β-actin antibody (Sigma, MO) at a dilution of 1∶200–3000. The secondary antibody was anti-mouse IgG, anti-rabbit IgG or anti-goat IgG (Amersham Biosciences, Piscataway, NJ and Santa Cruz Biotechnology Inc, CA) at a dilution of 1∶1000–3000. Immunoreactivity was detected as described before [29], [32].

### Gel Mobility Shifting Assays

Gel mobility shifting assays were conducted as previously described [33]–[34]. The following oligos were used: 5′-GTCCTTCATTACGTCACGCATAG-3′ for mouse PP2A-Aα promoter CREB binding site, 5′-GTCCTTCATTCATTCACGCATAG -3′ for mutated CREB binding site; 5′-TAAGATACTTCACTTCCGGTTC-3′ for mouse PP2A-Aα promoter ETS-1 binding site, 5′-TAAGATACTTCACTCAAGGTTC-3′ for mutated ETS-1 binding site; 5′- TCCGCCTCTCCCCAAGGGGCCATC-3′ for mouse PP2A-Aα promoter AP-2α (1) binding site, 5′-CTCCGCCTCTAAACAAGGGGCCATC-3′ for mutated AP-2α (1) binding site; 5′-CCGGCACCGCCCCGCCCGATC-3′ for mouse PP2A-Aα promoter SP-1 & SP-3 binding site, 5′-CCGGCACCGAACCGCCCGATC-3′ for mutated SP-1 & SP-3 binding site. Thirty µg of nuclear extracts prepared from ARPE, FHL124 or αTN4-1 cells were incubated with 1×10^5^ cpm of ^32^P-labeled double-stranded synthetic oligonucleotides for 30 minutes at 37°C in a binding shifting buffer [34]. For competition experiments, 50-fold of the non-labeled wild type or mutant double-stranded synthetic oligonucleotides were pre-incubated with the nuclear extracts for 10 minutes before the labeled probe was added into the reaction. For the supershifting experiments, 30 µg of each nuclear extract was incubated with 1×10^5^ cpm of ^32^P-labeled double-stranded synthetic oligonucleotides of each primer pair for 30 minutes at 37°C in a binding shifting buffer, then 10 µg antibody against CREB, ETS-1, AP-2α, SP-1, or SP-3 or normal IgG (mock) was incubated with the corresponding nuclear extract-primer complex for 45 minutes at room temperature. After the binding reactions, the mixtures were loaded onto 5% native PAGE and detected by autoradiography.

### Analysis of Transient Gene Expression

For reporter gene activity, 2 µg of A1 A2, A3, A4, A5 or A6 construct of the luciferase reporter gene and 20 ng internal control plasmid PhRL-sv40 were introduced into mouse αTN4-1 cells, human FHL124 cells, or human ARPE-19 cells in 12-well culture plates using lipofectamine 2000. After 24 hours, the luciferase activities from each testing construct (A1 A2, A3, A4, A5 or A6) and also from the internal control plasmid were measured using dual-luciferase reporter assay system from Promega Inc. The relative luciferase activity was determined by dividing the luciferase activity from the testing construct by that from the control plasmid. For CREB, ETS-1, AP-2α, SP-1 and SP-3 dose-dependent responses, 2 µg PP2A-Aα-luc construct plasmid (A5), and 20 ng internal control plasmid plus 0 to 500 ng of pCMV-CREB, pCMV-ETS-1, pCMV-AP-2α, pCMV-SP-1 or pCMV-SP-3 plasmid were co-transfected into both ARPE-19 and FHL124 cells, the transfected cells were harvested after 24 hours and the harvested cell extracts were used for assay of luciferase activity as described above.

### Chromatin Immunoprecipitation (ChIP) Assays

ChIP assay was conducted as previously described [34]. Mouse lens epithelial cells (αTN4-1) were grown to 95% confluence. Approximately 2.0×10^7^ cells were incubated with 1% formaldehyde for 10 min at room temperature for cross-linking, which was terminated by washing the cells with 4 ml of 1.25 M glycine solution. The cells were further washed with cold PBS twice and then scraped into 1 ml of ChIP sonication buffer (50 mM Tris-HCl, pH 8.1, 1% Triton X-100, 0.1% sodium deoxycholate, 5 mM EDTA, and 150 mM NaCl) containing the protease inhibitor cocktail. These lysates were sonicated 20–25 times for 10 s each time to generate DNA fragments that ranged in size from 200 to 1000 bp. The sheared chromatin-lysed extracts were incubated with 5 µg of anti-CREB, anti-ETS-1, anti-SP-1 or anti-AP-2α antibody separately or 5 µg of normal IgG overnight at 4°C, and then incubated for an additional 1 h with 30 µl protein A/G agarose beads. The immunoprecipitates were washed with cold ChIP sonication buffer 3X and cold PBS 3X, then suspended in the elution buffer (Tris-EDTA buffer, pH 8.0 and 1% SDS), and incubated overnight at 65°C, and an additional 2 h at 55°C with 100 µg of protease K to reverse proteins/DNA cross-links. Finally, these samples were processed for DNA purification by phenol-chloroform extraction and ethanol precipitation. PCRs were performed in 25 µl with 1/500 of input DNA or 1/100 of the immunoprecipitates with two pairs of primers: one for ETS-1 and CREB binding sites, 5′-GTCCTTCATTACGTCACGCATAG-3′ (forward), 5′-GAACCGGAAGTGAAGTATC-TTA -3′ (reverse); and the other for the AP-2α and SP-1 binding sites, 5′- TGGTTCAGACCAAACAGACG-3′ (forward), 5′-CTCCCTGGCGTTTCTATCAG-3′ (reverse); which generates DNA fragments of 189 bp and 168 bp, respectively. PCR was conducted with following specifications: 94°C 5 min, (94°C 30 s, 52°C 30 s, and 72°C 30 s)×30, 72°C 5 min, and the PCR products were separated on an 1.5% agarose gel and stained with ethidium bromide.

### Statistical Analysis

All of the data presented are derived from at least three independent experiments. All the luciferase reporter gene activity data were subjected to statistical analysis. The means, S.D. and P values were calculated and included in the corresponding figures.

## Results

### Isolation of the functional proximal PP2A–Aα promoter

**Figure 1:**
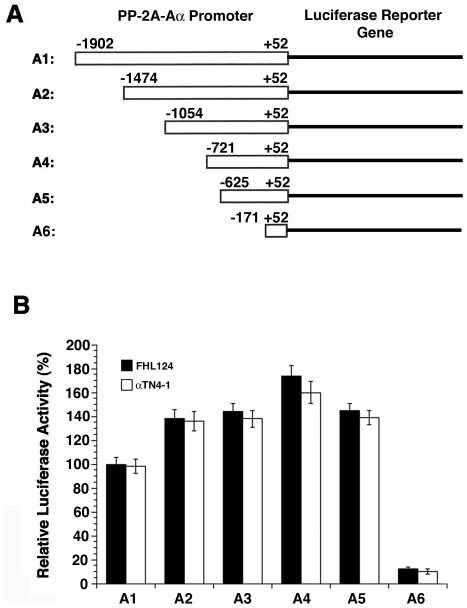
Identification of the proximal PP2A-Aα promoter. A. Diagrams of 6 different constructs of the PP2A-Aα promoter linked to the luciferase reporter gene. Genomic DNA was extracted from mouse muscle and used for PCR amplification of the PP2A-Aα promoter fragments (see methods for details). To identify the proximal promoter, 7 oligo primers (Table 1) were designed, synthesized and used for PCR. The amplified 6 DNA fragments were digested and then inserted into the basic luciferase reporter construct to generate A1 to A6 constructs. B. Relative luciferase activities derived from 6 different PP2A-Aα gene promoter constructs. Two µg of A1 A2, A3, A4, A5 or A6 construct of the luciferase reporter gene and 20 ng internal control plasmid PhRL-sv40 were introduced into mouse lens epithelial αTN4-1 cells (Open bars) and human lens epithelial FHL124 cells (solid bars) in 12-well culture plates using lipofectamine 2000. After 24 hours, the luciferase activities from each testing construct and also from the internal control plasmid were measured using dual-luciferase reporter assay system from Promega Inc. The relative luciferase activity was determined by dividing the luciferase activity from the testing construct by that from the control plasmid. The ratio of A1 activity is assumed as 100%, then the activity of each of the five constructs were calculated using A1 as reference. Note that the construct A5 displays similar luciferase activity to A3 and A4, suggesting that this construct contains the proximal promoter of the PP2A-Aα gene.

To dissect the proximal promoter for the PP2A-Aα gene, we amplified six genomic fragments of different sizes (Fig. 1A). These fragments were subsequently inserted into a basic luciferase reporter gene construct, pGL3-basic (from Promega) at Xho I (5′) and Hind III (3′) sites to make 6 luciferase reporter gene constructs, designated as A1 to A6 (Fig. 1A). Because PP-2A plays vital roles in the ocular tissues [30] and changed PP-2A activity is implicated in multiple ocular diseases including retina degeneration and lens cataract [25]–[26], we used retina and lens cell lines as assay systems to characterize the PP2A-Aα promoter. When the 6 constructs were individually transfected into mouse lens epithelial cells, αTN4-1 and human lens epithelial cells, FHL124, luciferase reporter gene activity assays demonstrated that compared with the longest construct A1, a sequential deletion from 5′ end of about 400 (A2) to 800 bp (A3) leads to about 40 to 50% enhancement of the luciferase activities, suggesting that a repressor element exists within this region (Fig. 1B). A deletion of additional 323 bp (A4) leads to further enhancement of the luciferase activity, indicating existence of additional repressor element within this region (Fig. 1B). Removal of 96 bp at 5′ end from A4 fragment yields construct A5 and causes a 20% decrease in luciferase activity, suggesting the presence of a positive enhancer from –721 to –625 (Fig. 1B). A further deletion of 500 bp at 5′ end from A5 fragment yielded construct A6, and caused a 80% drop of the luciferase activity (Fig. 1B). Since the luciferase activity driven by A5 is similar to that driven by A2 and A3 but drops significantly when driven by A6, we consider that the 677 bp PP2A-Aα promoter (−625 to +52) acts as the proximal promoter and was further examined in detail.

### The proximal PP2A–Aα promoter contains multiple putative *cis*-elements for CREB, ETS-1, AP-2α and SP-1

**Figure 2:**
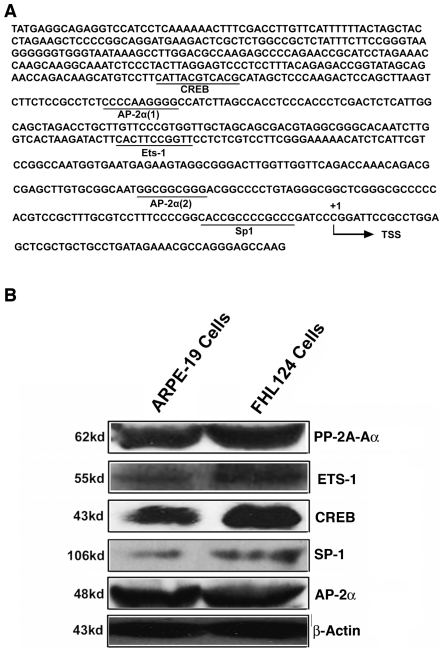
Identification of the major *cis*-elements in the proximal promoter of the PP2A-Aα gene. A. Identification of the four putative *cis*-elements: ETS-1, CREB, AP-2α and SP-1 in the proximal promoter of the PP2A-Aα gene. The 677 bp DNA sequence (from +52 to −625) was analyzed with TFSEARCH and TESS two softwares. Four conserved *cis*-elements (marked below the sequence) were discovered. In the core promoter region of the PP2A-Aα gene, a putative TFIIB recognition element (BRE) and a putative downstream promoter element (DPE) are observed. However, the core promoter of the PP2A-Aα gene lacks TATA-box. The transcription initiation site was assigned according to the reported results. B. Western blot analysis of PP2A-Aα and the four cognate transcriptional factors (ETS-1, CREB, AP-2α and SP-1) in two ocular cell lines, retinal epithelial cells (ARPE-19) and the embryonic lens epithelial cells (FHL124).

To characterize the PP2A-Aα gene promoter, we have analyzed the proximal promoter sequence using web software TFSEARCH (transcriptional factor search) and TESS (transcription element search system), and found four *cis*-elements with the highest scores for CREB, ETS-1, SP-1 and AP-2α in this region (Fig. 2A). The CREB binding site is found from −373 to −362, an ETS-1 binding site from −202 to −192, and two AP-2α binding sites from −321 to −312, and from −84 to −76, respectively (Fig. 2A). In addition, a SP-1 binding site is identified in the core promoter region from −17 to −6 of the PP2A-Aα gene. To explore if these elements might be functional in the ocular tissues, we first examined the association of the expressions of PP2A-Aα with that of the four transcription factors, CREB, ETS-1, SP-1 and AP-2α in the retinal pigmental epithelial cells (ARPE-19) and the embryonic lens epithelial cells (FHL124 cells). As shown in Fig. 2B, both cell lines contain relatively high levels of PP2A-Aα. Associated with the high level of PP2A-Aα expression, high levels of CREB and AP-2α expression are also detected in the two cell lines. In addition, fair levels of ETS-1 and SP-1 expression were also detected. Similar expression patterns of the above factors were observed in the mouse lens epithelial cells, αTN4-1 (data not shown). These results suggest that the four cis-elements, CREB, ETS-1, SP-1 and AP-2α in PP2A-Aα gene promoter are likely mediating transcription regulation by the cognate *trans*-factors in these ocular cell lines.

### ETS-1 interacts with the proximal promoter of PP2A-Aα and plays a fundamental role in PP2A-Aα regulation

**Figure 3:**
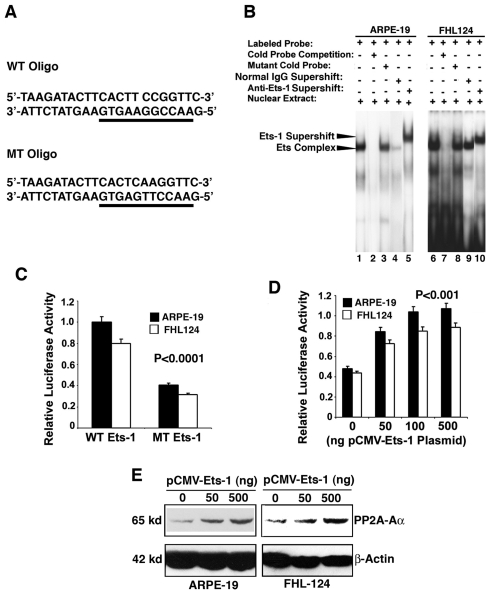
Demonstration that ETS-1 regulates the PP2A-Aα promoter. A. Wild type or mutant ETS-1 oligos used for gel mobility shifting assays described in Fig. 3B. B. Gel mobility shifting demonstrates that ETS-1 from both ARPE-19 (Lane 1 to 5) and FHL124 (Lane 6 to 10) binds to the oligo containing the wild type ETS-1 binding site. Nuclear extracts prepared from both types of cells were incubated with γ-^32^P-ATP-labeled oligos containing wild-type ETS-1 binding site under various conditions shown in the figure. The reaction mixtures were then separated with 5% native PAGE. The gel was dried and exposed to X-ray film for overnight. Lane 1, gel mobility shifting assays with labeled oligo containing wild-type ETS-1 binding site and ARPE-19 nuclear extract. Lane 2, the same assay as in lane 1 except that 50-fold of non-labeled wild-type ETS-1 oligos was added into the reaction. Note that the ETS-1 complex was competed off. Lane 3, the same assay as in lane 1 except that the non-labeled competing oligo contains a mutated ETS-1 binding site (Fig. 3A, bottom), which showed only slight competition ability. Lane 4, the same assay as in lane 1 except that normal IgG was used for mock supershifting assay. Lane 5, the same assay as in lane 1 except that anti-ETS-1 antibody was added for supershifting assay. Note that addition of anti-ETS-1 antibody into the reaction led to formation of the supershifting ETS-1 complex. Lane 6 to Lane 10, the same order as in Lane 1 to Lane 5 except that the nuclear extracts were from the FHL124 cells. C to E. Demonstration of the relative importance of the ETS-1 binding site in regulating the PP2A-Aα promoter. C. Mutation of the ETS-1 binding site in the PP2A-Aα promoter causes an approximately 60% loss of the luciferase reporter gene activity in both types of ocular cells. The P value was calculated by comparing the activity difference between the mutant promoter with the wild type promoter in the same type of cell. D. Expression of the exogenous ETS-1 in both types of ocular cells induces dose-dependent increase in the luciferase reporter gene activity within 0 to 500 ng of the pCMV-ETS-1 plasmid. The P value was calculated by comparing the luciferase difference between the vector (pCI-Neo) co-expression and the co-expression of each concentration of exogenous Ets-1 plasmid (50, 100 and 500 ng) with the wild type promoter in the same type of cell. E. Expression of the exogenous ETS-1 in both types of ocular cells enhanced expression of the endogenous gene coding for PP2A-Aα. Transfection and luciferase activity assays were conducted as previously described [29].

To demonstrate if ETS-1 regulates PP2A-Aα, we have conducted gel mobility shifting assay with an oligo containing the ETS-1 binding site (Fig. 3A), nuclear extracts from human ARPE-19 cells or from human FHL124 cells showed a strong binding to ETS-1 oligo (lane 1, 6 of Fig. 3B), this binding can be competed off by the cold ETS-1 oligo (lane 2 and 7 of Fig. 3B) but only slightly by the mutant oligo (lane 3 and 8 of Fig. 3B). The DNA-ETS-1 complex can be recognized by anti-ETS-1 antibody to form supershifting complex (lane 5 and 10 of Fig. 3B) but not by normal IgG (lane 4 and 9 of Fig. 3B). These results suggest that human ETS-1 could regulate mouse PP2A-Aα gene promoter in both ARPE-19 cells and FHL124 cells, and that the PP2A-Aα promoter may be functionally conserved between human and mouse. Similar binding pattern was observed with ETS-1 oligo and nuclear extract from mouse αTN4-1 cells (data not shown). To further confirm the ETS-1 control on PP2A-Aα gene, we mutated the ETS-1 binding site and examined the relative luciferase activities from the two constructs with either wild type or mutant ETS-1 binding sites in both ARPE-19 and FHL124 cells. As shown in Fig. 3C, mutation of the ETS-1 binding site led to about 60% decrease in the PP2A-Aα promoter activity as assayed in both types of cells. Next, we co-transfected the luciferase reporter gene driven by the PP2A-Aα proximal promoter and the exogenous ETS-1 expression vector. As shown in Fig. 3D, expression of the exogenous ETS-1 (0 to 100 ng) induced a dose-dependent increase in the luciferase activity in both ARPE-19 cells and FHL124 cells. Mutation of the ETS-1 binding site in the PP2A-Aα promoter abolished this dose-dependent response (data not shown). Further more, expression of the exogenous ETS-1 also enhanced expression of the endogenous PP2A-Aα (Fig. 3E). Together, these results demonstrate that ETS-1 can regulate PP2A-Aα and this regulation is very important. In addition, the PP2A-Aα promoter is functionally conserved between human and mouse.

### CREB binds to the proximal promoter of PP2A-Aα and plays an important regulatory role in PP2A-Aα expression

**Figure 4:**
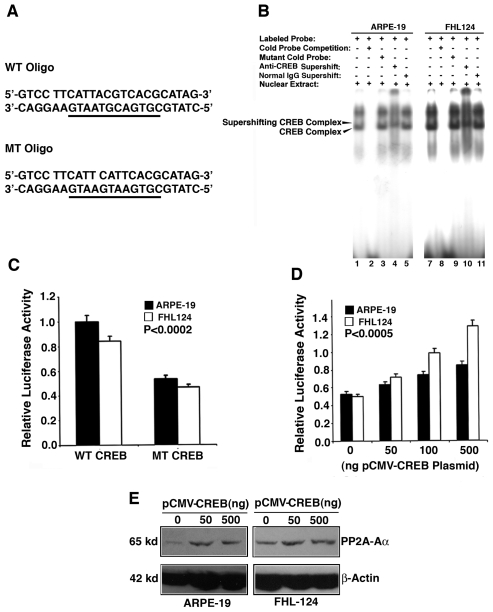
Demonstration that CREB regulates the PP2A-Aα promoter. A. Wild type or mutant CREB oligos used for gel mobility shifting assays described in Fig. 4B. B. Gel mobility shifting demonstrates that CREB from both ARPE-19 (Lane 1 to 5) and FHL124 (Lane 6 to 10) nuclear extracts binds to the oligo containing the wild type CREB binding site. Nuclear extracts prepared from both types of cells were incubated with γ-^32^P-ATP-labeled oligos containing wild-type CREB binding site under various conditions shown in the figure. Lane 1, gel mobility shifting assay with labeled wild type CREB oligo and ARPE-19 nuclear extract. Lane 2, the same assay as in lane 1 except that 50-fold of wild-type CREB non-labeled oligos was added into the reaction. Note that the CREB complex was competed off. Lane 3, the same assay as in lane 1 except that the non-labeled competing oligo contains a mutated CREB binding site (Fig. 4A, bottom), which showed very little competition ability. Lane 4, the same assay as in lane 1 except that anti-CREB antibody was added into the reaction. Note that addition of anti-CREB antibody into the reaction led to formation of the supershifting CREB complex. Lane 5, the same assay as described in lane 1 except that normal IgG was added into the reaction to conduct mock supershifting assay. Lane 6 to Lane 10, the same order as in Lane 1 to Lane 5 except that the nuclear extracts were from the FHL124 cells. C. to E. Demonstration of the relative importance of the CREB binding site in regulating the PP2A-Aα promoter. C. Mutation of the CREB binding site in the PP2A-Aα promoter causes an approximately 40% loss of the luciferase reporter gene activity in both types of ocular cells. The P value was calculated by comparing the activity difference between the mutant promoter with the wild type promoter in the same type of cell. D. Expression of the exogenous CREB in both types of ocular cells induces dose-dependent increase in the luciferase reporter gene activity within 0 to 500 ng of the pCMV-CREB plasmid. The P value was calculated by comparing the luciferase difference between the vector (pCI-Neo) co-expression and the co-expression of each concentration of exogenous CREB plasmid (50, 100 and 500 ng) with the wild type promoter in the same type of cell. E. Expression of the exogenous CREB in both types of ocular cells enhanced expression of the endogenous gene coding for PP2A-Aα. Transfection and luciferase activity assays were conducted as previously described [29].

To determine if the CREB element can regulate PP2A-Aα promoter, we also conducted gel mobility shifting assay with an oligo primer containing the CREB binding site derived from the PP2A-Aα promoter (Fig. 4A). The nuclear extracts from either ARPE-19 cells or from FHL124 cells showed a strong binding to CREB site (lane 1, 6 of Fig. 4B), this binding can be competed off by the cold CREB oligo (lane 2, 7 of Fig. 4B) but not by the mutant oligo (lane 3, 8 of Fig. 4B). Addition of the anti-CREB antibody into the binding reactions between labeled CREB oligo and the nuclear extracts from both ARPE-19 and FHL124 cells induced obvious supershifting complex (lane 4, 9 of Fig. 4B). However, addition of normal IgG into the same reactions did not produce the supershifting complex (lane 5, 10 of Fig. 4B). Similar binding pattern was observed with CREB oligo and nuclear extract from mouse αTN4-1 cells (data not shown). These results suggest that CREB also regulates PP2A-Aα. To further confirm that CREB controls the PP2A-Aα gene promoter, we mutated the CREB binding site and compared the relative luciferase activity between the two constructs with wild type or mutant CREB binding sites in both ARPE-19 and FHL124 cells. As shown in Fig. 4C, mutation of the CREB binding site led to about 40% decrease in the PP2A-Aα promoter activity as assayed in both types of cells. Thus, reporter gene activity assays on wild type PP2A-Aα promoter and differentially mutated PP2A-Aα promoters reveal that ETS-1 and CREB display differential control over PP2A-Aα promoter. Next, we also conducted co-transfection analysis on the luciferase reporter gene activity driven by the A5 promoter with exogenous CREB expression construct. As shown in Fig. 4D, expression of the exogenous CREB (0 to 500 ng) induces a dose-dependent increase in the luciferase activity in both ARPE-19 cells and FHL124 cells. Moreover, the magnitude of induction is greater in FHL124 cells than in ARPE-19 cells. Mutation of the CREB site in the PP2A-Aα promoter abolished this dose-dependent response (data not shown). In addition, expression of the exogenous CREB also enhanced expression of the endogenous PP2A-Aα in both types of ocular cells (Fig. 4E). Together, these results show that CREB also plays an important role in regulating PP2A-Aα activity, and further demonstrate that the PP2A-Aα promoter is functionally conserved in human and mouse.

### AP-2α interacts with the proximal promoter of PP2A-Aα and exerts positive control

**Figure 5:**
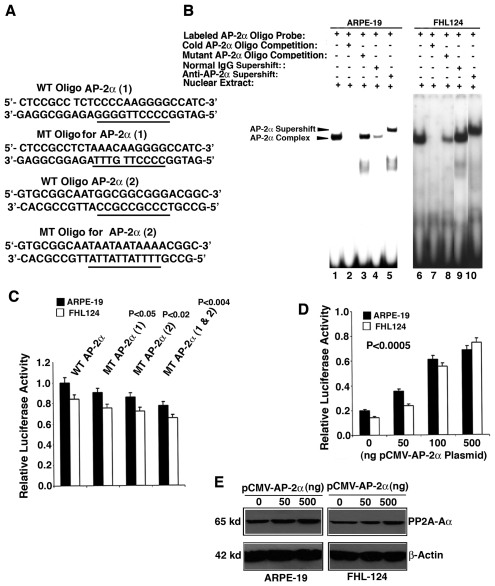
Demonstration that AP-2α regulates the PP2A-Aα promoter. A. Wild type or mutant AP-2α (1) oligos used for gel mobility shifting assays described in Fig. 5B. B. Gel mobility shifting demonstrates that AP-2α from both ARPE-19 and FHL124 nuclear extracts binds to the oligo containing the wild type AP-2α (1) binding site. Nuclear extracts prepared from ARPE-19 cells (Lane 1 to 5) and FHL124 cells (Lane 6 to 10) were incubated with γ-^32^P-ATP-labeled oligo-nucleotide containing wild-type AP-2α binding site under various conditions shown in the figure. The reaction mixtures were then separated with 5% native PAGE. The gel was dried and exposed to X-ray film for overnight. Lane 1, gel mobility shifting assays with labeled oligo containing wild-type AP-2α binding site and nuclear extract from ARPE-19 cells. Lane 2, the same assay as described for lane 1 except that 50-fold of non-labeled oligo containing the wild-type AP-2α binding site was added into the reaction. Note that the AP-2α complex was competed off by the non-labeled oligo. Lane 3, the same assay as described in lane 1 except that the non-labeled competing oligo contains a mutated AP-2α binding site (Fig. 5A, bottom), which showed much weaker competition ability. Lane 4, the same assay as described in lane 1 except that normal IgG was added into the reaction to conduct mock supershifting assay. Lane 5, the same assay as described in lane 1 except that anti-AP-2α antibody was added into the reaction. Note that addition of anti-AP-2α antibody into the reaction led to formation of the supershifting AP-2α complex. Lane 6 to Lane 10, the same order as in Lane 1 to Lane 5 except that the nuclear extracts were from the FHL124 cells. C. to E. Demonstration of the relative importance of the AP-2α binding site in the regulation of the PP2A-Aα promoter. C. Mutations of the AP-2α (1), AP-2α (2) or both AP-2α sites in the PP2A-Aα promoter cause approximately 10%, 15% and 18% reduction in the luciferase reporter gene activity, respectively. The P value was calculated by comparing the activity difference between each individual mutant promoter with the wild type promoter, or between the mutant promoter with both AP-2α sites mutated with the wild type promoter in the same type of cell. D. Expression of the exogenous AP-2α in both types of ocular cells induces dose-dependent increase in the luciferase reporter gene activity within 0 to 500 ng of the pCMV-AP-2α plasmid. The P value was calculated by comparing the luciferase difference between the vector (pCI-Neo) co-expression and the co-expression of each concentration of exogenous AP-2α plasmid (50, 100 and 500 ng) with the wild type promoter in the same type of cell. E. Expression of the exogenous AP-2α in both types of ocular cells only slightly enhanced expression of the endogenous gene coding for PP2A-Aα. Transfection and luciferase activity assays were conducted as previously described [29].

To test if AP-2α also regulates PP2A-Aα gene promoter, we again conducted gel mobility shifting assay with an oligo containing the AP-2α (1) binding site (Fig. 5A). The nuclear extracts from either ARPE-19 cells or from FHL124 cells showed a strong binding to AP-2α (1) site (lane 1, 6 of Fig. 5B), this binding can be completely competed off by the cold AP-2α (1) oligo (lane 2, 7 of Fig. 5B) but only partially by the mutant oligo {lane 3, 8 of Fig. 5B, A change of 3-nucleatide in the AP-2α (1) binding site only partially abolished the binding activity}. Addition of the anti-AP-2α antibody into the binding reactions between labeled AP-2α oligo and the nuclear extracts from the two types of cells induced a supershifting band (lane 5, 10 of Fig. 5B). This supershifting complex did not appear when the normal IgG was used (lane 4, 9 of Fig. 5B). Similar binding pattern was observed with AP-2α oligo and nuclear extract from mouse αTN4-1 cells (data not shown). These results suggest that AP-2α also regulates PP2A-Aα To further confirm the AP-2α control on PP2A-Aα gene promoter, we again mutated the two AP-2α binding sites either individually or in combination (the mutation of the two AP-2α sites are shown in Fig. 5A) and assayed the relative luciferase activity from the two constructs in both ARPE-19 and FHL124 cells. As shown in Fig. 5C, mutation of each AP-2α binding site led to 10 to 15% decrease in the PP2A-Aα promoter activity as assayed in both types of cells. Mutation of both AP-2α sites at the same time caused an approximately 20% decrease in the reporter gene activity. Statistical analysis revealed that the P value is less than 0.05 when each mutant promoter was compared with the wild type promoter. Thus, mutation of either AP-2α site causes statistically significant change in the PP2A-Aα promoter activity (Fig. 5C). Next, we examined the luciferase reporter gene activity in the presence of the exogenous AP-2α expression. As shown in Fig. 5D, expression of the exogenous AP-2α (0 to 500 ng) induced a dose-dependent increase in the luciferase activity in both ARPE-19 cells and FHL124 cells. Mutations of the two AP-2α binding sites in the PP2A-Aα promoter abolished this dose-dependent response (data not shown). In addition, expression of the exogenous AP-2α also enhanced expression of the endogenous PP2A-Aα in both types of ocular cells (Fig. 5E). Together, these results show that AP-2α displays a positive regulation on the PP2A-Aα promoter.

### The interaction between SP-1/SP-3 and the proximal promoter of PP2A-Aα leads to negative regulation

**Figure 6:**
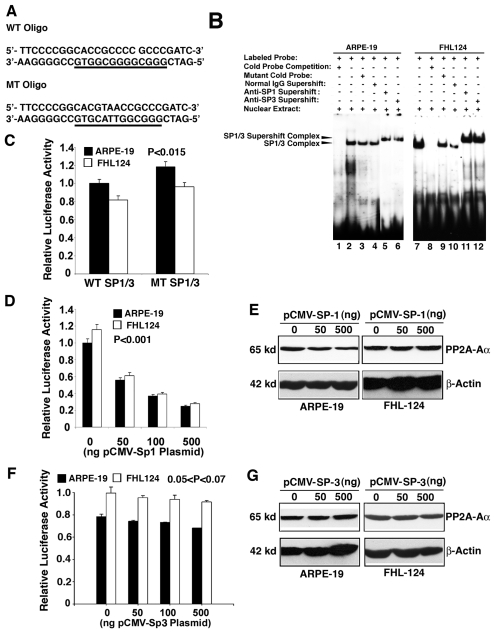
Demonstration that SP-1 and SP-3 negatively regulates the PP2A-Aα promoter. A. Wild type or mutant SP-1/SP-3 oligos used for gel mobility shifting assays described in Fig. 6B. B. Gel mobility shifting demonstrates that SP-1/SP-3 from both ARPE-19 and FHL124 nuclear extracts binds to the oligo containing the wild type SP-1/SP-3 binding site. Nuclear extracts prepared from ARPE-19 cells (Lane 1 to 6) and FHL124 cells (Lane 7 to 12) were incubated with γ-^32^P-ATP-labeled oligo-nucleotide containing wild-type SP-1/SP-3 binding site under various conditions shown in the figure. The reaction mixtures were then separated with 5% native PAGE. The gel was dried and exposed to X-ray film for overnight. Lane 1, gel mobility shifting assays with labeled oligo containing wild-type SP-1/SP-3 binding site and nuclear extract from ARPE-19 cells and 50-fold of non-labeled oligo containing the wild-type SP-1/SP-3 binding site. Note that the SP-1/SP-3 complex was competed off by the non-labeled oligo. Lane 2, gel mobility shifting assays with labeled oligo containing wild-type SP-1/SP-3 binding site and nuclear extract from ARPE-19 cells. Lane 3, the same assay as described in lane 2 except that the non-labeled competing oligo contains a mutated SP-1/SP-3 binding site (Fig. 6A, bottom), which showed much weaker competition ability. Lane 4, the same assay as described in lane 2 except that normal IgG was added into the reaction to conduct mock supershifting assay. Note that addition of normal IgG interferes with the formation of the SP-1/SP-3 complex but did not change the mobility. Lane 5, the same assay as described in lane 2 except that anti-SP-1 antibody was added into the reaction. Note that addition of anti-SP-1 antibody into the reaction led to formation of the supershifting SP-1 complex. Lane 6, the same assay as described in lane 2 except that anti-SP-3 antibody was added into the reaction. Note that addition of anti-SP-3 antibody into the reaction also led to formation of the supershifting SP-3 complex. Lane 7, gel mobility shifting assays with labeled oligo containing wild-type SP-1/SP-3 binding site and nuclear extract from FHL124 cells; Lane 8, the same as lane 7 except that 50-fold of non-labeled oligo containing the wild-type SP-1/SP-3 binding site was added into the reaction. Note that the SP-1/SP-3 complex was completely competed off by the non-labeled oligo. Lane 9 to Lane 12, the same order as in Lane 3 to Lane 6 except that the nuclear extracts were from the FHL124 cells. C to G. Demonstration of the relative importance of the SP-1/SP-3 binding site in the regulation of the PP2A-Aα promoter. C. Mutation of the SP-1/SP-3 binding site in the PP2A-Aα promoter causes an approximately 17 to 18% enhancement of the luciferase reporter gene activity in both types of ocular cells. The P value was calculated by comparing the activity difference between the mutant promoter with the wild type promoter in the same type of cell. D. Expression of the exogenous SP-1 in both types of ocular cells induces dose-dependent decrease in the luciferase reporter gene activity within 0 to 500 ng of the pCMV-SP-1 plasmid. The P value was calculated by comparing the luciferase difference between the vector (pCI-Neo) co-expression and the co-expression of each concentration of exogenous SP-1 plasmid (50, 100 and 500 ng) with the wild type promoter in the same type of cell. E. Expression of the exogenous pCMV-SP-1 in both types of ocular cells attenuated expression of the endogenous gene coding for PP2A-Aα. F. Expression of the exogenous SP-3 in both types of ocular cells induced only slight decrease in the luciferase reporter gene activity within 0 to 500 ng of the pCMV-SP-3 plasmid, which is not statistically significant. The P value was calculated by comparing the luciferase difference between the vector (pCI-Neo) co-expression and the co-expression of each concentration of exogenous SP-3 plasmid (50, 100 and 500 ng) with the wild type promoter in the same type of cell. G. Expression of the exogenous pCMV-SP-3 in both types of ocular cells caused little change in the expression of the endogenous gene coding for PP2A-Aα. Transfection and luciferase activity assays were conducted as previously described [29].

To elucidate if SP-1/SP-3 regulates PP2A-Aα promoter, we also conducted gel mobility shifting assay with an oligo containing the SP-1/SP-3 binding site from PP2A-Aα (Fig. 6A). The nuclear extract from ARPE-19 cells displayed a relatively weak binding to SP-1/SP-3 oligo (lane 2 of Fig. 6B) compared with the extract from FHL124 cells (lane 7 of Fig. 6B). Nevertheless, this binding is specific in both types of cells because it can be completely competed off by the cold SP-1/SP-3 oligo (lane 1, 8 of Fig. 6B) but much less competition (lane 9 of Fig. 6B) or very little competition (lane 3 of Fig. 6B) was observed with the mutant oligo. Addition of the anti-SP-1 antibody (lane 5 and 11 of Fig. 6B) or anti-SP-3 antibody (lane 6 and 12 of Fig. 6B) but not normal IgG (lane 4 and 10 of Fig. 6B) into the binding reactions between labeled SP-1/SP-3 oligo and the nuclear extracts from the two types of cells induced the supershifting band. Similar binding pattern was observed with SP-1/SP-3 oligo and nuclear extract from mouse αTN4-1 cells (data not shown). These results suggest that SP-1/SP-3 also regulates PP2A-Aα gene. To determine the nature of the SP-1/SP-3 regulation on PP2A-Aα gene, we mutated the SP-1/SP-3 binding site within the core promoter of PP2A-Aα, and compared the relative luciferase activity between the two constructs with either wild type or mutant SP-1/SP-3 binding sites in both ARPE-19 and FHL124 cells. As shown in Fig. 6C, mutation of the SP-1/SP-3 binding site led to 17 to 18% increase in the PP2A-Aα promoter activity as assayed in both types of cells. The P value for this difference is less than 0.015 when the mutant promoter was compared with the wild type promoter in the same type of cell assayed. Thus, mutation of SP-1/SP-3 site causes statistically significant change (Fig. 6C). To confirm this negative regulation, we also conducted co-transfection analysis of the luciferase reporter gene activity in the presence of the exogenous SP-1 expression (Fig. 6D) or exogenous SP-3 expression (Fig. 6F). As shown in Fig. 6D, expression of the exogenous SP-1 induces a dose-dependent decrease in the luciferase activity in both ARPE-19 cells and FHL124 cells, and this down-regulation is statistically significant (P<0.001). Mutation of the SP-1 site in the PP2A-Aα promoter abolished this dose-dependent response (data not shown). On the other hand, expression of the exogenous SP-3 induced a slight decrease in the luciferase activity in both ARPE-19 cells and FHL124 cells, which is not statistically significant since the P value is larger than 0.05. In addition, expression of the exogenous SP-1 led to some down-regulation in the expression of the endogenous PP2A-Aα in both types of ocular cells (Fig. 6E). But expression of the exogenous SP-3 caused very little change in the expression of the endogenous PP2A-Aα in both types of ocular cells (Fig. 6G). Thus, different from ETS-1 CREB and AP-2α, SP-1 exerts negative control over PP2A-Aα promoter and SP-3 displays some negative effect which is not statistically significant.

### ChIP assays reveal that ETS-1, CREB, AP-2α and SP-1 all binds to PP2A-Aα promoter

**Figure 7:**
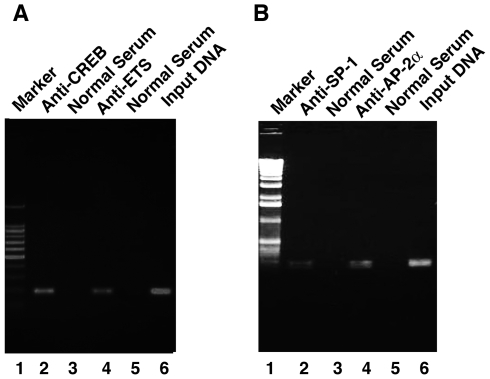
ChIP assays to demonstrate that ETS-1, CREB, AP-2α and SP-1 bind to PP-2α promoter. ChIP assays were conducted as we recently described [34]. The oligos used for ChIP assays were described in Materials and Methods. A. ChIP assays to show that ETS-1 and CREB bind to PP-2Aα promoter. Lane 1, DNA marker; lane 2, PCR product derived from 1/50 DNA template immunoprecipitated by anti-CREB antibody; Lane 3, PCR product derived from DNA template immunoprecipitated by normal IgG; lane 4, PCR product derived from 1/50 DNA template immunoprecipitated by anti-ETS-1 antibody; Lane 5, PCR product derived from DNA template immunoprecipitated by normal IgG; lane 6, PCR product derived from direct input DNA template without immunoprecipitation. A band of 189 bp containing both CREB and ETS-1 binding sites in the PP2A-Aα promoter gene was amplified. ChIP assay confirms that CREB and ETS-1 bind to PP2A-Aα gene. B. ChIP assays to show that AP-2α and SP-1 bind to PP-2Aα promoter. Lane 1, DNA marker; lane 2, PCR product derived from 1/50 DNA template immunoprecipitated by anti-SP-1 antibody; Lane 3, PCR product derived from DNA template immunoprecipitated by normal IgG; lane 4, PCR product derived from 1/50 DNA template immunoprecipitated by anti-AP-2α antibody; Lane 5, PCR product derived from DNA template immunoprecipitated by normal IgG; lane 6, PCR product derived from direct input DNA template without immunoprecipitation. A band of 168 bp containing both AP-2α and SP-1 binding sites in the PP2A-Aα promoter gene was amplified. ChIP assays confirm that AP-2α and SP-1 bind to PP2A-Aα gene.

To further confirm that the four *cis*-elements found in the proximal promoter of PP2A-Aα were functional in vivo, we conducted ChIP assays using mouse αTN4-1 cells [34]. In these cells, a high level of PP2A-Aα expression was also detected (data not shown). When the endogenous PP2A-Aα gene chromatin from αTN4-1 cells were randomly fragmented through sonication, antibody against ETS-1, CREB, SP-1 or AP-2alpha used for immunoprecipitation was able to precipitate down the PP2A-Aα proximal promoter sequences which can be amplified into 189 bp (lane 2, 4 of Fig. 7A) or 168 bp (lane 2, 4 of Fig. 7B) fragment after PCR reaction. As a comparison, the immunoprecipitated products using normal IgG did not contain the PP2A-Aα promoter DNA sequences (lane 3, 5 of Fig. 7A and 7B). As positive controls, the isolated genomic DNA input was directly used for PCR analysis and the corresponding 189 bp and 168 bp fragments were also identified (lane 6 of Fig. 7A and 7B). These results suggest that the four *cis*-elements identified in the PP2A-Aα promoter play important roles in mediating transcriptional regulation by the corresponding transcriptional factors.

## Discussion

**Figure 8:**
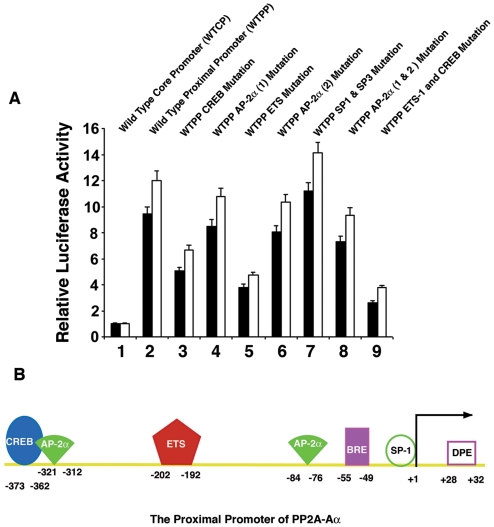
Summary of the PP2A-Aα promoter. A. Relative importance of various *cis*-elements in the PP2A-Aα promoter as demonstrated by the differential reporter gene activities driven by wild type or various mutant PP2A-Aα promoter. 1: The wild type PP2A-Aα core promoter containing BRE, DPE, AP-2α (2) and the SP-1 binding site (A6); 2: The wild type PP2A-Aα proximal promoter containing CREB, ETS-1, AP-2α(1), AP-2α (2), BRE, DPE and SP-1 binding sites (A5); 3: The PP2A-Aα proximal promoter containing CREB, ETS-1, AP-2α(1), AP-2α (2), BRE, DPE and SP-1 binding sites with CREB binding site mutated; 4: The PP2A-Aα proximal promoter containing CREB, ETS-1, AP-2α(1), AP-2α (2), BRE, DPE and SP-1 binding sites with AP-2α(1) binding site mutated; 5: The PP2A-Aα proximal promoter containing CREB, ETS-1, AP-2α(1), AP-2α (2), BRE, DPE and SP-1 binding sites with ETS-1 binding site mutated; 6: The PP2A-Aα proximal promoter containing CREB, ETS-1, AP-2α(1), AP-2α (2), BRE, DPE and SP-1 binding sites with AP-2α(2) binding site mutated; 7: The PP2A-Aα proximal promoter containing CREB, ETS-1, AP-2α(1), AP-2α (2), BRE, DPE and SP-1 binding sites with SP-1 binding site mutated; 8: The PP2A-Aα proximal promoter containing CREB, ETS-1, AP-2α(1), AP-2α (2), BRE, DPE and SP-1 binding sites with AP-2α(1) and AP-2α(2) binding sites mutated; 9: The PP2A-Aα proximal promoter containing CREB, ETS-1, AP-2α(1), AP-2α (2), BRE, DPE and SP-1 binding sites with CREB and ETS-1 binding sites mutated. B. Diagram to show the relative positions of the cis-elements for general transcriptional factors and also the four major cis-elements for the cognate transcription factors in the PP2A-Aα proximal promoter.

In the present study, we have conducted functional dissection of the PP2A-Aα gene promoter and obtained the following results: 1) Sequential deletion and luciferase reporter gene activity assays revealed that the mouse proximal promoter of PP2A-Aα consists of about 680 bp DNA fragment; 2) Four major *cis*-elements for ETS-1, CREB, AP-2α and SP-1/SP-3 are present in the proximal promoter; 3) DNA binding assays demonstrate that human ETS-1, CREB, AP-2α, SP-1 and SP-3 bind to the corresponding four *cis*-elements within the mouse proximal promoter of PP2A-Aα, and the proximal promoter of PP2A-Aα is functionally conserved in human and mouse; 4) In vitro mutagenesis and luciferase reporter gene activity assays demonstrate that ETS-1, CREB, and AP-2α act as enhancers and SP-1 and SP-3 as repressors on PP2A-Aα promoter in both human retinal pigment epithelial cells and lens epithelial cells; 5) Expression of exogenous ETS-1, CREB, AP-2α, or SP-1 induced dose-dependent responses of the luciferase reporter gene activity and also similarly regulates the endogenous gene for PP2A-Aα in human retinal pigmental epithelial cells and embryonic lens epithelial cells. Mutation of the corresponding cis-element eliminated the related dose-dependent response; 6). ChIP assays revealed that ETS-1, CREB, AP-2α or SP-1 all bind to the proximal promoter of PP2A-Aα. Together, our results have demonstrated that the proximal promoter of PP2A-Aα is regulated by four major *cis*-elements through interactions with their cognate transcriptional factors besides the basic core promoter elements mediating interactions with the general transcription factors (Fig. 8).

### The proximal promoter of PP2A-Aα consists of BRE, DPE, and multiple enhancer elements but lack TATA element

Previous studies of different eukaryotic gene promoters reveal that a typical core promoter contains the TFIIB recognition element {BRE element (GGGCGCC)} at −37 to −26, the TATA box (TATAAA) at −31 to −26, the initiator (PyPyANT/ApyPy) at −2 to +4, and the downstream promoter element {DPE element (A/GGA/TC/TG/A/C)} at +28 to +32 [35]. Examination of the core promoter for PP2A-Aα reveals the presence of the putative BRE element (GGGCGCC) at the −55 to −49, and the putative DPE element (TGATA) at +28 to +32 (Fig. 2A). The PP2A-Aα promoter, however, lacks the typical TATA box. The presence of the putative DPE element allows the binding of the general transcriptional factor, TFIID as found in most TATA-less promoter [36]–[39]. Moreover, the putative BRE element allows the binding of TFIIB [40]–[41]. In the core promoter, an additional element, the SP-1/SP-3 binding site, was identified from −6 to −18. The binding of SP-1/SP-3 to this region of the promoter likely interferes with the binding of the general transcription factors. Indeed, in vitro mutagenesis and luciferase reporter gene activity assays revealed that this SP-1 binding site exerts negative control (Fig. 6C and 6D), which is different from its action in other target genes [42]. Introduction of the exogenous SP-1 expression vector into both retinal pigment epithelial cells and lens epithelial cells induces dose-dependent decrease of the PP2A-Aα promoter activity and also downregulates expression of the endogenous PP2A-Aα gene in these cells, further confirming that the SP-1 binding site within the core promoter acts as a repressor (Fig. 6). Introduction of the exogenous SP-3 expression vector into the two types of cells also lead to negative control, which seems not statistically significant.

Outside of the core promoter of the PP2A-Aα gene, three well-conserved *cis*-elements are identified: one ETS-1 binding site localized from −202 to −192, one CREB binding site found from −373 to −362, and two AP-2α binding sites localized from −321 to −312, and from −84 to −76 (Fig. 2A). Depending on the target genes and interacting partners, CREB, ETS-1 and AP-2α may act as either positive regulators or repressors [43]–[46]. In our study, gel mobility shifting assays with nuclear extracts from ARPE-19 or FHL124 cells reveal that both ETS-1 and CREB can strongly bind to the cognate *cis*-elements, indicating that these *cis*-elements are functional. *In vitro* mutagenesis and reporter gene assays demonstrate that both ETS-1 and CREB binding sites in the proximal promoter of the PP2A-Aα play very important role. Mutations of the ETS-1 and CREB sites individually lead to a loss of 58% and 43% promoter activity, respectively (Fig. 3C and 4C). Mutation of both sites at the same time causes additional lose of the proximal PP2A-Aα promoter, suggesting that both ETS-1 and CREB have some synergistic effects (Fig. 8A). Besides the ETS-1 and CREB binding sites, there are two copies of AP-2α binding sites in the proximal promoter. The AP-2α (2) site localized between −84 to −76 is more conserved than the other AP-2α (1) site localized within −321 to −312 (Fig. 2A). Consistent with this conservation is the effect they displayed on the reporter gene activity. When mutated, the promoter with the mutant AP-2α (2) loses about 15% promoter activity. In contrast, mutation of the AP-2α (1) causes about 10% lose in the promoter activity (Fig. 6B). Mutation of both AP-2α sites leads to a total of 18% loss in the PP2A-Aα promoter activity, also indicating the partial synergistic action between the two AP-2α sites (Figs. 6C and 8A). In both retinal epithelial cells and human lens epithelial cells, the enhancement of transcription by ETS-1, CREB, and AP-2α was further confirmed by the co-transfection study in which a dose-dependent response of the reporter gene activity was observed for each factor (Fig. 3D, 4D and 5D). Moreover, expression of each exogenous transcription factor also displays similar mode of action to the endogenous PP2A-Aα gene (Fig. 3E, 4E, and 5E). Thus, in vitro mutagenesis and co-transfection assays demonstrate that all three *cis*-elements act as enhancers in the PP2A-Aα promoter. This conclusion is further confirmed by ChIP assays (Fig. 7). Together, our results demonstrate that the PP2A-Aα promoter, though lacking the TATA box, contains the putative BRE and DPE elements mediating the basic regulation by the general transcriptional factors [35] and the enhancer elements (ETS, CREB and AP-2α) mediating the advanced control by the cognate *trans*-factors.

### PP2A-Aα/β Plays A Critical Role in the Assembly of the Functional PP-2A

As a major eukaryotic phosphatase, the normal function of PP-2A is essential in maintaining the organism homeostasis and preventing various pathological conditions such as cancer [6], [18]–[22]. PP-2A exists as either a heterodimeric core enzyme including the scaffold A subunit and catalytic C subunit, or a heterotrimeric holoenzyme consisting of the core enzyme plus one of the regulatory B subunits, thus providing temporal and spatial specificity of PP-2A activity within tissue cells of different organisms.

Since both C and B subunits bind to the A subunit, the normal function of the scaffold A subunit plays a critical role in the assembly of either core enzyme or holoenzyme of PP-2A to govern its specific activity. This conclusion is derived from numerous studies. First, interruption of the function of the scaffold subunit by the small t antigen inhibits PP2A activity. The virus-encoded small t antigen (ST) of DNA tumor viruses SV40 and polyomavirus can exclusively bind to PP2A-Aα/β [47]–[48] in the HEAT repeats 3 to 6 [49]–[50]. As a result of this binding, the phosphatase activity of the PP-2A core enzyme, but not the holoenzyme, was inhibited by the t antigen [51]. Reudiger et al. [52] have shown that varying the ratio of PP-2A core enzyme to holoenzyme causes significant biological consequence. Binding of the t antigen to PP2A-Aα/β stimulates MAPK activation and induces cell proliferation [53] and eventually cell transformation [54].

Second, mutations in the gene encoding PP2A-Aα leads to abolished PP-2A activity. Calin et al. [19] described four cancer-associated mutations in the PP2A-Aα gene: Glu64-Asp in lung carcinoma, Glu64-Gly in breast carcinoma, Arg418-Trp in melanoma, and a deletion mutant missing residue 171 to residue 589 in breast carcinoma. Reudiger et al. [22] have shown that these mutations greatly interrupt the interactions of the scaffold subunit with either B subunit alone (Glu64-Asp and Glu64-Gly), or with both B and C subunits (Arg418–Trp, and the deletion mutant), thus abolishing specific PP-2A activity and leading to tumor formation.

In addition, the normal expression level of PP2A-Aα gene plays an essential role in the assembly of functional PP-2A. It has been shown that both A and C subunits are expressed in similar levels in normal cells [22]. However, in about 43% brain tumor patients (Gliomas), PP-2A activity was significantly lower than that found in the normal brain tissue [23]. This decrease in PP-2A activity is neither derived from changed expression of the catalytic subunit of PP-2A nor from mutations of the PP2A-Aα/β subunits but a 10-fold downregulation in the expression of PP2A-Aα [23]. What accounts for the downregulation of PP2A-Aα in these brain tumors remains to be further explored. Nevertheless, our demonstrations that the proximal promoter of PP2A-Aα contains multiple *cis*-elements including the binding sites for ETS-1, CREB, AP-2α and SP-1, and that these *cis*-elements are all functional provide some clues. The downregulated expression of the PP2A-Aα gene found in the gliomas [23] may be derived from the changed expression levels and functions of the related transcription factors. In this regard, it is worth to mention that ETS-1 is closely involved in gliomas development. ETS-1 protein is not only differentially expressed in astrocytes and astrocytoma cells [55] but also regulates various targets such as Egr-1, cathepsin B and the urokinase-type plasminogen activator besides PP2A-Aα in gliomas [56]. A reduced expression of PP2A-Aα has also been observed in other cancer cells such as breast cancer MCF-7 cells [24] and prostate cancer cells (Li et al. unpublished data). Our preliminary studies suggest the reduced PP2A-Aα expression is derived from changed expression levels of one or more transcription factors.

In summary, our characterization of the PP2A-Aα promoter lays a foundation for the further exploration on why PP2A-Aα is differentially expressed in the different types of cancers. Elucidation of the regulatory mechanisms governing PP2A-Aα expression will contribute fundamental knowledge to the understanding of the PP-2A functions in carcinogenesis and also other human diseases.
